# The development and validation of the Cluster Headache Quality of life scale (CHQ)

**DOI:** 10.1186/s10194-016-0674-1

**Published:** 2016-09-05

**Authors:** Norazah Abu Bakar, Mariam Torkamani, Surat Tanprawate, Giorgio Lambru, Manjit Matharu, Marjan Jahanshahi

**Affiliations:** 1 Headache Group, UCL Institute of Neurology and The National Hospital for Neurology and Neurosurgery, Queen Square, London, WC1N 3BG UK; 2 Cognitive Motor Neuroscience Group, Sobell Department of Motor Neuroscience & Movement Disorders, UCL Institute of Neurology and The National Hospital for Neurology and Neurosurgery, Queen Square, London, WC1N 3BG UK

**Keywords:** Cluster headache, Trigeminal autonomic cephalalgia, Headache, Quality of life

## Abstract

**Background:**

Cluster headache (CH) is a rare, excruciating and highly disabling primary headache disorder. Using non cluster headache specific measures, previous studies have shown that CH has a significant negative impact on patients’ quality of life (QoL), but a CH-specific QoL scale is currently unavailable. Thus, the objective of this study was to develop and validate a CH-specific QoL scale.

**Methods:**

Based on a literature review, semi-structured patient interviews and expert panel consultation, we produced a 54-item questionnaire, which was pre-tested in a sample of CH patients and subsequently reduced to 47 items. The revised scale was then administered to CH sufferers attending a tertiary headache clinic and those registered with a patient group. A total of 406 completed questionnaires were received. To assess test-retest reliability, a subsample (*N* = 56) completed the scale on a second occasion, two weeks after the first. Standard statistical methods were used to analyse the data for validity and reliability.

**Results:**

Item reduction and exploratory factor analysis led to 28-items, grouped into four subscales labelled “restriction of activities of daily living”, “impact on mood and interpersonal relationships”, “pain and anxiety”, and “lack of vitality”. The final CH-specific QoL scale, the CHQ, demonstrated satisfactory internal consistency (Cronbach’s alpha > 0.9) and test-retest reliability (intraclass correlation coefficient > 0.8), with good internal construct validity between subscales (range 0.52–0.75) and convergent validity with other QoL measures.

**Conclusions:**

We have developed and validated the first patient-reported outcome measure of QoL specifically for CH sufferers, which may be used to monitor QoL in clinical care and research.

## Background

Quality of life (QoL) scales have increasingly emerged as an important clinical outcome measure for assessing the impact of a disorder and its treatment on patients’ wellbeing [3, 18]. Within the headache field, much of the interest in this area has been focused on migraine, due to its high prevalence. A number of disease-specific QoL instruments have been developed for migraine [11, 14, 17, 23]. Similar measures for other primary headache types, such as cluster headache, are not yet available.

Cluster headache (CH) is a rare, excruciating and highly disabling headache that is strictly lateralised, typically associated with prominent cranial autonomic features or a sense of restlessness or agitation [10, 12]. In CH, assessment of QoL is currently limited to use of generic scales, such as the SF-36, and headache disability instruments, which have shown significantly diminished scores compared to headache-free controls [2, 5, 7, 16, 21]. Moreover, a study found significant differences between CH patients in the ictal versus the interictal period, but no significant differences between CH patients and migraineurs [7]. The authors postulated that since the study used a migraine-specific measure, it might not have been able to truly capture the essential aspects of CH [7]. In light of this and the differences between the two headache entities, we aim to develop a CH-specific QoL tool, which may better reflect the true nature of the daily life impact of this highly disabling disorder. We report the development and validation of a disease-specific QoL instrument for CH.

## Methods

Ethical approval for this study was obtained from The North West London Research Ethics Committee (Date of ethics approval: 26 July 2010, Ethics ID number: 10/H0722/43). Informed written consent was obtained from all participants prior to enrolment in the study. Data was collated in an electronic database and all statistical analyses were performed using SPSS-PASW software version 18. A three-step approach was employed in the development and validation of the scale: first item generation; second item reduction and scale development; and, finally scale validation and reliability testing.

### Item generation

A comprehensive review of the literature was conducted and existing headache-specific QoL scales were studied to generate an overview of the areas of life impacted by CH. This was followed by an in-depth semi-structured interview of 24 episodic and chronic CH patients in 2010 (M:F 2.6:1, mean age 46.3 years), diagnosed according to the diagnostic criteria of the International Classification of Headache Disorders (ICHD-II) [12], who are registered with the headache clinic at The National Hospital for Neurology and Neurosurgery, London and were living in and around the Greater London area. The topics covered during the interview included pain characteristics, aspects of the patient’s life that were affected by their headaches, their support system, and their outlook on life. These processes allowed generation of a preliminary questionnaire, which was then discussed with a panel of experts with an interest in headache. Any ambiguous or similar items were eliminated or grouped together, before a final set of items were agreed upon. A 54-item questionnaire was subsequently drafted, each with a range of five possible answers on a Likert scale: never, occasionally, sometimes, often and always, addressing areas of life impacted by CH within the past month or during their last cluster bout. A visual analogue scale (VAS) was added to the end of the questionnaire to rate overall satisfaction with life (0 = extremely dissatisfied, 100 = extremely satisfied). Subsequently, a pilot study was conducted with 24 CH patients to assess the face validity and clarity, and the questionnaire was then adjusted accordingly and reduced to 47-items.

### Item reduction and scale development

There were two sources of CH participants for this study: (i) Patients with a diagnosis of CH attending the headache clinic at The National Hospital for Neurology and Neurosurgery, London and (ii) an invitation letter to participate in the survey was posted out to CH participants via OUCH UK (The Organisation for the Understanding of Cluster Headache, United Kingdom). Those who responded to the invitation letter were contacted via telephone and had their headaches phenotyped via a telephone interview. Inclusion criteria for the study were those who had a clinical diagnosis of CH, whilst the exclusion criteria were those who had other major neurological, psychiatric or physical illness. A booklet of questionnaires, which included the 47-item CH-QoL questionnaire was then given or posted out to all participants who satisfied the ICHD-II diagnostic criteria for CH (*n* = 521) from 2011 to 2013. Details on demographics, headache history and characteristics were collected from the questionnaires. A number of other frequently utilised generic or headache specific QoL instruments were also included in the booklet to allow assessment of convergent validity of this new scale, including the SF-36 Health Survey Questionnaire, the EuroQoL (EQ-5D) Questionnaire and the Migraine-Specific Quality of Life Questionnaire Version 2.1 (MSQ v2.1).

The SF-36 Health Survey Questionnaire is a generic QoL measure with excellent reliability and validity [24]. It contains 36 self-administered items, measuring functions in eight domains; physical functioning (PF), role-physical (RP), bodily pain (BP), general health (GH), vitality (VT), social role functioning (SF), emotional role functioning (EF) and mental health (MH). The subscales are scored on a scale of 0 to 100, with higher scores indicating better QoL in the domain being measured [24].

The EuroQoL (EQ-5D) Questionnaire is a generic measure of current health status. It consists of five domains: mobility, self-care, usual activities, pain/discomfort and anxiety/depression. In addition, there is a visual analogue scale, with 0 being the worst imaginable and 100 being the best imaginable current health state [19].

The Migraine-Specific Quality of Life Questionnaire Version 2.1 (MSQ v2.1) is a 14-item measure specifically developed to assess the QoL in patients with migraine. The items are divided across three domains; role restrictive, role preventive and emotional functioning. This questionnaire has been shown to have good internal consistency and construct validity [17]. The total possible score ranges from 14 to 84, with higher scores indicating poorer QoL.

A total of 406 completed questionnaires were received, giving a response rate of 77.9 %. From this total, 36.5 % were recruited from the headache clinic and 63.5 % were recruited from OUCH UK. About fifty-nine percent of the responders had episodic CH and 41.1 % had chronic CH. The mean age of the study sample was 52.4 years (range 20.5–84.4). There were 68.2 % males and 31.8 % females, with a mean age of onset of CH of 33.0 years (range 8.0–69.0).

Intercorrelation between variables was performed on the data generated from the survey. Items that showed low intercorrelations (*r* < 0.1) were excluded as this demonstrated that they were poorly correlated with the underlying scale. On the other hand, any items that showed high intercorrelations (*r* > 0.7) [6] were examined and the least clinically sensible item was excluded, as theoretically items that correlated too highly are measuring the same underlying dimension [8].

### Scale validation and reliability testing

Construct validity was assessed with an exploratory factor analysis to determine the key components of the 47-item questionnaire. Oblique rotation was used for the analysis, as we had reason to believe that the resulting factors would correlate with each other. An eigenvalue cut off point >1 was used to extract underlying factors. Concurrent validity was assessed by measuring the Pearson’s correlation of the underlying subscales of the CH-specific HRQoL questionnaire with the subscales of SF-36 and MSQ v2.1. Meanwhile, Spearman’s correlation test was employed to assess validity of the questionnaire and the EQ-5D, due to the ordinal nature of the latter.

A second copy of the 47-item questionnaire was sent out to 75 respondents (approximately 15 % of the main validation sample size) two weeks after the first completion of it to allow assessment of the test-retest reliability of the new scale. Fifty-six completed questionnaires were received (71.7 % response rate) for the assessment of test-retest reliability. The mean age of this subsample was 55.7 years (range 34.7–79.1). There were 66.1 % males and 33.9 % females, with a mean age of onset of CH of 36.4 years (range 12.0–66.0).

## Results

### Participants

There were no significant differences in the sociodemographic and headache characteristics of the participants based on the source of their recruitment, as shown in Table 1. Moreover, no significant differences were found in the CH-specific HRQoL scores between males and females.

**Table 1:** Sociodemographic and headache characteristics of the participants based on method of recruitment

Characteristic	Total (*n* = 406)	NHNN (*n* = 148)	OUCH UK (*n* = 258)	*p* values*
Age, mean ± SD	52.4 ± 12.3	51.1 ± 12.1	53.2 ± 12.4	0.109
Gender (male: female)	2.1: 1	2.2: 1	2.1: 1	0.820
Marital status, *n* (%)				0.079
Single	60 (14.9 %)	25 (16.9 %)	35 (13.7 %)	
Married/cohabiting	304 (75.2 %)	103 (69.6 %)	201 (78.5 %)	
Widowed	12 (3.0 %)	4 (2.7 %)	8 (3.1 %)	
Divorced/separated	28 (6.9 %)	16 (10.8 %)	12 (4.7 %)	
Years of education, *n* (%)				0.122
1–11	109 (27.4 %)	45 (31.0 %)	64 (25.3 %)	
12–13	73 (18.3 %)	32 (22.1 %)	41 (16.2 %)	
14–17	167 (42.0 %)	55 (37.9 %)	112 (44.3 %)	
18 +	49 (12.3 %)	13 (9.0 %)	36 (14.2 %)	
Smokers, *n* (%)	186 (45.8 %)	70 (47.2 %)	116 (45.0 %)	0.649
Duration since onset of CH (years), mean ± SD	19.3 ± 11.5	18.8 ± 10.7	19.6 ± 12.0	0.483
Duration of CH (minutes), mean ± SD	46.5 ± 58.8	48.6 ± 52.9	45.2 ± 62.1	0.601
Frequency of CH attacks (number/day), mean ± SD	3.5 ± 2.4	3.5 ± 2.1	3.5 ± 2.5	0.892
Severity of CH attacks, *n* (%)				0.057
Mild	1 (0.2 %)	1 (0.7 %)	0 (0.0 %)	
Moderate	15 (3.7 %)	9 (6.1 %)	6 (2.3 %)	
Severe	29 (7.1 %)	15 (10.1 %)	14 (5.4 %)	
Very severe	61 (15.0 %)	20 (13.5 %)	41 (15.9 %)	
Excruciating	300 (73.9 %)	103 (69.6 %)	197 (76.4 %)	

*NHNN* The National Hospital for Neurology and Neurosurgery, *OUCH UK* The Organisation for the Understanding of Cluster Headache, United Kingdom, *CH* cluster headache, *SD* standard deviation

*Based on two-sample *t*-tests for continuous variables and Chi-square tests for categorical variables

### Construct validity

The exploratory factor analysis produced five factors, consisting of 37 items. One factor with an eigenvalue of 1.11 (explaining 3.1 % of the variance) was removed as it only had one item loading onto it and therefore was considered insufficient to produce a meaningful subscale [22]. The remaining factors and items were then examined to determine if there was any scope for further reduction of the number of items to produce a more meaningful and user-friendly scale. Eight items were omitted as they failed to gain significant loading (>0.4) on any of the factors created. This resulted in a 28-item questionnaire (CHQ), which explained 56.1 % of the variance (Table 2). The Cronbach’s alpha was calculated and compared for the factors prior to, and after removal of these items, to ensure that it did not compromise the internal consistency of the scale [4]. Expert opinion was also sought throughout this process to ensure there was no removal of clinically relevant items.

**Table 2:** Results of the principal component factor analysis of the CHQ scale

Items	Factor 1 Restriction of ADL	Factor 2 Impact on mood and interpersonal relationships	Factor 3 Pain and anxiety	Factor 4 Lack of vitality
Avoided leaving the house	0.76			
Avoided making plans due to unpredictability of CH e.g., holidays	0.72			
Felt unable to complete duties at work	0.66			
Had difficulty in getting involved in leisure activities e.g., cinema, theatre, etc.?	0.63			
Avoided crowded and noisy places e.g., public transport, pubs, etc.	0.57			
Felt that the severity of cluster headache affected your daily activities	0.56			
Been less involved in family affairs e.g., interaction with children, planning holidays	0.54			
Been unable to socialise/spend time with friends and family	0.46			
Been unable to achieve your daily goals and carry out routines and chores	0.42			
Felt less respected by others		0.89		
Had problems with close personal relationship		0.73		
Felt you were a burden on family and friends		0.71		
Felt self-conscious and uncomfortable about your appearance after a cluster headache attack (e.g., swelling/redness of eyes and facial sweating, etc.)		0.68		
Felt that others are dismissive of your cluster headaches		0.61		
Felt aggressive		0.53		
Felt bad about yourself, lost self-confidence or felt worthless		0.53		
Felt like harming yourself or suicidal		0.53		
Been irritable, impatient or less tolerant		0.53		
Been forgetful e.g., missed appointments		0.49		
Been unable to take care of your appearance (e.g., take a bath, put make-up on, change clothes etc.)		0.49		
Felt isolated, lonely or vulnerable		0.45		
Found your pain is unbearable if untreated			0.67	
Dreaded that the headache would not go away			0.48	
Felt lacking in energy and constantly tired				−0.88
Felt sleepy, worn out or less able to concentrate due to nocturnal attacks of CH				−0.72
Had problems concentrating e.g., reading paper, watching TV, etc.				−0.62
Been unable to think clearly				−0.60
Felt tense or anxious				−0.48
% of variance explained	43.11	5.59	4.06	3.38

*CHQ* cluster headache specific quality of life, *ADL* activities of daily living

Based on the results of the factor analysis, nine items were grouped onto a factor addressing various ‘*Restrictions of activities of daily living’* (ADL), such as avoiding leaving the house, making plans and inability to complete duties at work. Twelve items described *‘Impact on mood and interpersonal relationships’*, such as feelings of being dismissed by others and worthlessness, including any suicidal tendencies. Two items loaded on a ‘Pain and anxiety’ factor, which addressed the pain of the cluster headache and any associated anxiety such as dreading that the headache not going away. Finally, a ‘*Lack of vitality’* (five items) factor addresses problems related to energy and cognition, for example difficulties in thinking clearly and concentration. There was good intercorrelation between the subscales (range 0.52–0.75) derived from the factor analysis, supporting internal construct validity. There was also a moderate correlation between the total score and the VAS (*r* = −0.57, *p* < 0.01). This negative correlation was expected as their scores ran in opposite direction (higher VAS indicates better HRQoL whereas higher total score indicates poorer HRQoL).

### Internal consistency and test-retest reliability

The scale had a Cronbach’s coefficient alpha (α) of 0.95, which was well above the recommended criteria of 0.70. The internal consistency and corrected item to total correlations of the items to their resulting subscale is shown in Table 3. Test-retest reliability testing of the scales was performed on the data collected from respondents who completed the questionnaire on two occasions, which showed significant correlation between the two assessment occasions (intra class correlation coefficient was 0.87). Cronbach’s alpha was also satisfactory for the scales on both occasions (Table 4).

**Table 3:** Item to total correlations and Cronbach’s alpha for the CHQ scale

Scale/Items	Corrected item to total correlation	Cronbach’s alpha
Restriction of ADL		0.91
Avoided leaving the house	0.67	
Avoided making plans due to unpredictability of CH e.g., holidays	0.66	
Felt unable to complete duties at work	0.70	
Had difficulty in getting involved in leisure activities e.g., cinema, theatre, etc.?	0.78	
Avoided crowded and noisy places e.g., public transport, pubs, etc.	0.63	
Felt that the severity of cluster headache affected your daily activities	0.68	
Been less involved in family affairs eg interaction with children,planning holidays	0.67	
Been unable to socialise/spend time with friends and family	0.73	
Been unable to achieve your daily goals and carry out routines and chores	0.69	
Impact on mood and interpersonal relationships		0.90
Felt less respected by others	0.72	
Had problems with close personal relationship	0.71	
Felt you were a burden on family and friends	0.73	
Felt self-conscious and uncomfortable about your appearance after a cluster headache attack (eg swelling/redness of eyes and facial sweating, etc.)	0.65	
Felt that others are dismissive of your cluster headaches	0.50	
Felt aggressive	0.59	
Felt bad about yourself, lost self-confidence or felt worthless	0.63	
Felt like harming yourself or suicidal	0.59	
Been irritable, impatient or less tolerant	0.63	
Been forgetful e.g., missed appointments	0.58	
Been unable to take care of your appearance (eg take a bath, put make-up on, change clothes etc.)	0.55	
Felt isolated, lonely or vulnerable	0.71	
Pain and anxiety		0.52
Found your pain is unbearable if untreated	0.37	
Dreaded that the headache would not go away	0.37	
Lack of vitality		0.85
Felt lacking in energy and constantly tired	0.69	
Felt sleepy, worn out or less able to concentrate due to nocturnal attacks of CH	0.61	
Had problems concentrating e.g., reading paper, watching TV, etc.	0.64	
Been unable to think clearly	0.70	
Felt tense or anxious	0.66

**Table 4:** Cronbach’s alpha and test-retest reliability at the first and second assessment occasions

Scale	Cronbach’s alpha time 1	Cronbach’s alpha time 2	Test-retest reliability
Restriction of ADL	0.91	0.93	0.84 *p* < 0.01
Impact on mood and interpersonal relationships	0.90	0.87	0.83 *p* < 0.01
Pain and anxiety	0.52	0.63	0.71 *p* < 0.01
Lack of vitality	0.85	0.80	0.80 *p* < 0.01

### Convergent validity

The scale was assessed for convergent validity by measuring correlation of the subscales with the relevant subscales of the EQ-5D, SF-36 and MSQ (Table 5). With regards to the EQ-5D, the CHQ subscales showed low to moderate correlations with all of the EQ-5D domains, except for the pain and anxiety subscale of CHQ and the mobility and self-care domains (EQ-5D). The highest correlation observed was between the ‘Impact on mood and interpersonal relationships’ subscale of the CHQ and anxiety/depression item of the EQ-5D (*r*_*s*_ = 0.54, *p* < 0.01).

**Table 5:** Correlation coefficients (*p* values) between the CHQ subscales and the EQ-5D and SF-36 generic and MSQ v2.1 migraine-specific quality of life measures

	Restriction of ADL	Impacts on mood and interpersonal relationships	Pain and anxiety	Lack of vitality
EQ-5D				
Mobility	0.28^a^	0.27^a^	0.07^c^	0.21^a^
Self-care	0.31^a^	0.39^a^	0.10^b^	0.27^a^
Usual activities	0.35^a^	0.43^a^	0.14^a^	0.29^a^
Pain/discomfort	0.19^a^	0.30^a^	0.11^b^	0.14^a^
Anxiety/depression	0.39^a^	0.54^a^	0.28^a^	0.38^a^
SF-36				
Physical functioning (PF)	−0.27^a^	−0.37^a^	−0.11^b^	−0.24^a^
Role physical (RP)	−0.23^a^	−0.32^a^	−0.05^c^	−0.19^a^
Bodily pain (BP)	−0.23^a^	−0.31^a^	−0.12^b^	−0.22^a^
General health (GH)	−0.34^a^	−0.46^a^	−0.15^a^	−0.32^a^
Vitality (VT)	−0.37^a^	−0.49^a^	−0.22^a^	−0.43^a^
Social functioning (SF)	−0.47^a^	−0.52^a^	−0.21^a^	−0.37^a^
Role emotional (RE)	−0.41^a^	−0.50^a^	−0.21^a^	−0.39^a^
Mental health (MH)	−0.51^a^	−0.67^a^	−0.37^a^	−0.50^a^
MSQ v2.1				
Role restrictive (RR)	0.51^a^	0.53^a^	0.29^a^	0.45^a^
Role preventive (RP)	0.56^a^	0.52^a^	0.28^a^	0.42^a^
Emotional functioning (EF)	0.49^a^	0.65^a^	0.41^a^	0.47^a^

^a^ *p* < .01: ^b^ *p* < .05: ^c^non-significant

Similarly, there were low to moderate correlations with the SF-36 and MSQ subscales. The ‘Restrictions of ADL’ factor of CHQ correlated significantly with the social role functioning (SF) (*r* = −0.47, *p* < 0.01) and emotional role functioning (RE) subscales (*r* = −0.41, *p* < 0.01). The ‘Impact on mood and interpersonal relationships’ subscale of the CHQ correlated highly with mental health (MH) (*r* = −0.67, *p* < 0.01) and moderately with SF (*r* = −0.52, *p* < 0.01), RE (*r* = −0.50, *p* < 0.01) and vitality (VT) (*r* = −0.49, *p* < 0.01) subscales. The ‘Lack of vitality’ subscale of the CHQ correlated moderately with VT (*r* = −0.43, *p* < 0.01) and RE (*r* = −0.39, *p* < 0.01). All the correlations were negative as the CHQ and SF-36 were scored in different directions. In relation to the MSQ, the CHQ subscales correlated significantly with the all domains. Furthermore, the ‘Impact on mood and interpersonal relationships’ subscale of the CHQ and emotional functioning domain of the MSQ were well correlated (*r* = 0.65, *p* < 0.01), supporting good convergent validity.

The mean scores of the CHQ in episodic CH patients were then compared to those of chronic CH patients. Patients with chronic CH had significantly greater impairment in their QoL compared to their episodic counterparts (61.6 ± 18.9 vs 56.2 ± 19.0, *p* = 0.010), further supporting good validity and sensitivity of the questionnaire to detect impairment specifically related to CH.

## Discussion

Several studies have demonstrated that QoL is significantly impaired in patients with CH, more so in chronic sufferers, with considerable impact on daily living e.g., efficiency and ability to work and social functioning, with almost 20 % of patients losing their jobs secondary to the disorder [5–7, 13, 15, 20, 21]. However, these studies have all used either generic QoL scales such as the SF-36, or migraine-specific scales that may not necessarily be able to capture the true effects of CH, and may therefore be underestimating the actual impact of the disorder on QoL. Indeed, some instruments specifically ask about suffering in the past four weeks, thus ECH sufferers out of a bout would rate low scores on these scales, even though they may be severely impaired during a bout [9]. Hence, these measures may not provide a true reflection of the actual impairment. Moreover, issues that are specific to CH are not addressed through the use of these scales, for example suicidal tendencies, which is prevalent among CH sufferers. Circadian periodicity is another distinct feature in this disorder, with sufferers usually being woken up around the same time every night, at the onset of rapid eye movement (REM) sleep, which can have a major impact on patients with CH. We have therefore developed a questionnaire based on both patient and expert views of the disorder. To our knowledge, this is the first scale developed to measure QoL specifically in CH sufferers.

In the current study, we developed and validated a CH-specific QoL scale, the CHQ. Items for the scale were generated from an in-depth literature review and semi-structured patient interviews, allowing CH sufferers to express their views about the various aspects of their lives that they felt were affected by the disorder and should be highlighted in such a disease specific QoL scale. This was followed by a review by a panel of experts with an interest in headaches to include items that were considered clinically relevant. These steps allowed us to develop a scale that is based on both patient and clinician input, thus ensuring good content and face validity. Furthermore, following administration to a large sample of participants with CH, the scale has also been shown to have good construct validity, internal consistency and test-retest reliability. In terms of convergent validity, the subscale scores showed good correlation with those of other widely used QoL scales that have already been shown to have good validity and reliability, specifically the EQ-5D, SF-36 and MSQ. Moreover, the scale has been able to detect significantly greater impairment in QoL in chronic CH patients, compared to episodic CH patients, thus showing good sensitivity.

A limitation of this study was that about a third of our study population comprised patients attending a tertiary referral centre; hence medically intractable cases may be over-represented in our sample. Of the 148 patients recruited from the headache clinic, 64.2 % had chronic CH, which is significantly greater than is expected in the general population [1]. Thus our sample may not be totally representative of the CH population in the community. However, this bias enabled us to collect data from a fair proportion of chronic CH sufferers (42.9 %), who due to the recurring nature of their headaches are likely to be more disabled by this disorder, giving us a better picture of the extent of the impact on patients QoL. Since the development of the questionnaire involved both CH patients and a panel of experts, we strongly believe that the items included in our questionnaire were equally important for both ECH and CCH sufferers. Furthermore, despite the different methods of recruitment, it could be argued that those recruited via OUCH UK were also significantly affected by their CH to warrant them to seek help, as shown by the lack of significant differences in their headache characteristics from those recruited through the hospital, hence, supporting our decision to group them together.

## Conclusions

We have developed the first objective measure of QoL specifically for CH sufferers, which we have intended to be brief and user-friendly as it takes about 10 min to complete the questionnaire. We hope that it can be used in the clinical setting to monitor QoL as part of patient care, as well as in clinical trials as a patient-reported outcome measure. The next stage in the validation of the CHQ will be an assessment of its sensitivity to capture change in QoL over time (e.g., in the active and remission phases in episodic CH) and following medical and surgical treatments of CH. Further studies will also need to be performed in other community populations as the development and validation of this scale was based solely on a sample of CH population in the United Kingdom. Future studies could also examine age or gender related differences in QoL in CH with the CHQ. Once the CHQ has been fully validated and its sensitivity to change established, it could serve as an appropriate measure for identifying the demographic (e.g., age, gender, socioeconomic status), clinical (e.g., severity of pain, frequency of episodes, distribution of pain), psychological (mood, anxiety), social (e.g., social support) and behavioural (e.g., ways of coping, avoidance) factors that predict QoL in CH. Such information would be valuable in steering clinical management to focus on aspects of the disease that would help enhance QoL of CH sufferers.

## Abbreviations

ADL: Activities of daily living
BP: Bodily pain
CCH: Chronic cluster headache
CH: Cluster headache
CHQ: Cluster headache specific quality of life
ECH: Episodic cluster headache
EF: Emotional role functioning
EQ-5D: The European Quality of Life scale
GH: General health
HRQoL: Health related quality of life
ICHD: International Classification of Headache Disorders
MH: Mental health
MSQ: Migraine-Specific Quality of Life Questionnaire
OUCH-UK: The Organisation for the Understanding of Cluster Headache, United Kingdom
PF: Physical functioning
QoL: Quality of life
REM: Rapid eye movement
RP: Role-physical
SF: Social role functioning
SF-36: The Short Form 36-items Health Survey
SPSS-PASW: Statistical Package for the Social Sciences-Predictive Analytics Software
VAS: Visual analogue scale
VT: Vitality
